# Progress in promoting data sharing in public health emergencies

**DOI:** 10.2471/BLT.17.192096

**Published:** 2017-04-01

**Authors:** Katherine Littler, Wee-Ming Boon, Gail Carson, Evelyn Depoortere, Sophie Mathewson, Daniel Mietchen, Vasee S Moorthy, Denise O’Connor, Cathy Roth, Carlos Segovia

**Affiliations:** a Wellcome Trust, Gibbs Building, 215 Euston Road, London NW1 2BE, England.; b Research Policy and Translation, National Health and Medical Research Council, Melbourne, Australia.; c Nuffield Department of Medicine, University of Oxford, Oxford, England.; d Directorate-General for Research and Innovation, European Commission, Brussels, Belgium.; e National Library of Medicine, National Institutes of Health, Bethesda, United States of America.; f Information, Evidence and Research, World Health Organization, Geneva, Switzerland.; g Department for International Development, the Government of the United Kingdom of Great Britain and Northern Ireland, London, England.; h Directorate for Research Evaluation and Promotion, Instituto de Salud Carlos III, Madrid, Spain.; Correspondence to Katherine Littler (email: K.Littler@wellcome.ac.uk).

In February 2016, the World Health Organization (WHO) declared the Zika virus-related cluster of microcephaly cases and other neurological disorders reported in Brazil, a Public Health Emergency of International Concern (PHEIC).^1^ Following the declaration, over 30 global health bodies issued a joint statement committing to data sharing to ensure that the global response to the Zika virus and future emergencies, could be informed by the best and most current evidence.^2^ The statement represented a concerted effort by those involved to address past failures of timely access to relevant data. It also highlighted the lack of a clear path to implementation for data sharing during public health emergencies. In March 2016, the Global Research Collaboration for Infectious Disease Preparedness (GloPID-R) established a data-sharing working group which has been working in coalition with other stakeholders including**WHO, scientists, nongovernmental organizations, journals and other agencies.^3^ This group is working to identify barriers to data sharing in public health emergencies that should be addressed to better prepare for any future epidemic. We review the progress since the joint statement was made, outline the key challenges related to data sharing and summarize the group’s activities to date.

The experiences from the 2013–2016 Ebola virus disease outbreak and the 2015 Zika virus outbreak demonstrated the importance of research in public health emergencies and the difficulties associated with sharing research findings rapidly and outside of conventional scientific publications.^4^^–^^7^ Research – whether epidemiological, genetic, preclinical, microbiological, behavioural or operational – can generate new knowledge about an outbreak in rapidly changing situations. Research can inform risk communication, surveillance, clinical care, product development and other interventions. The WHO consensus and policy statements called for a paradigm shift in information sharing in public health emergencies and described the particularities to consider in dealing with different data types.^8^^,^^9^

Despite these efforts, rapid data sharing during public health emergencies remains challenging for various reasons. First, there are limited incentives for researchers and other people responding to the emergency to share data. Second, there is a lack of appropriate infrastructure for data sharing such as repositories and information technology platforms. Such rapid data sharing requires a clear governance structure that ensures a balance between privacy and access, as well as adheres to national and international ethical and legal requirements. Implementation of calls for data sharing is hampered by barriers, including: (i) inequity in capacity and funding between researchers in high-and low-income settings; (ii) varying concepts of data ownership by data providers and data users; (iii) no clear mechanism for attribution and academic recognition for data providers and data users related to published products; (iv) costs and varying degrees of access to data management systems within research groups or institutions; (v) reputational risk from premature sharing of data and results; (vi) ethical and regulatory issues related to privacy and consent in the context of experimental treatment and clinical care; (vii) access to the benefits of research; (viii) concerns about loss of potential financial benefits from eventual commercialization and intellectual property rights.^10^

The GloPID-R working group has developed, and requests comment on, a set of principles to underpin future implementation of timely data sharing.^11^ These new principles draw on others, such as the *FAIR Guiding Principles for scientific data management and stewardship*,^12^ and are intended to provide an initial framework for discussion. The group is also preparing case studies to document data-sharing practices in past emergencies; developing a decision tool to guide data sharing to address knowledge gaps in outbreaks and has commissioned studies on good practice and standards. The intention is to use the emerging evidence base to inform the design and implementation of new systems and approaches that address the data needs of the different groups responding to public health emergencies. The collective work is intended to support WHO’s *Research and Development Blueprint* and include other stakeholders, such as the Global Outbreak Alert and Response Network and the Coalition for Epidemic Preparedness. 

Effective data sharing requires flexibility by all stakeholders to adapt to unforeseen events and challenges. A data-sharing system needs to allow collaboration between stakeholders in the absence of pre-existing relationships and all collaborators need to adhere to fundamental ethical principles of data use. Above all, it must ensure that people in all affected countries benefit from timely access to evidence-based interventions in emergencies.
